# Transcriptomic analysis provides insights into molecular mechanisms of thermal physiology

**DOI:** 10.1186/s12864-022-08653-y

**Published:** 2022-06-04

**Authors:** Melissa K. Drown, Douglas L. Crawford, Marjorie F. Oleksiak

**Affiliations:** University of Miami Rosenstiel School of Marine and Atmospheric Science, 6400 Rickenbacker Causeway, Miami, FL USA

**Keywords:** Metabolism, Cardiac metabolism, Critical thermal maximum, Evolutionary adaptation, Co-expression network analysis

## Abstract

Physiological trait variation underlies health, responses to global climate change, and ecological performance. Yet, most physiological traits are complex, and we have little understanding of the genes and genomic architectures that define their variation. To provide insight into the genetic architecture of physiological processes, we related physiological traits to heart and brain mRNA expression using a weighted gene co-expression network analysis. mRNA expression was used to explain variation in six physiological traits (whole animal metabolism (WAM), critical thermal maximum (CT_max_), and four substrate specific cardiac metabolic rates (CaM)) under 12 °C and 28 °C acclimation conditions. Notably, the physiological trait variations among the three geographically close (within 15 km) and genetically similar *F. heteroclitus* populations are similar to those found among 77 aquatic species spanning 15–20° of latitude (~ 2,000 km). These large physiological trait variations among genetically similar individuals provide a powerful approach to determine the relationship between mRNA expression and heritable fitness related traits unconfounded by interspecific differences. Expression patterns explained up to 82% of metabolic trait variation and were enriched for multiple signaling pathways known to impact metabolic and thermal tolerance (*e.g.*, AMPK, PPAR, mTOR, FoxO, and MAPK) but also contained several unexpected pathways (*e.g.*, apoptosis, cellular senescence), suggesting that physiological trait variation is affected by many diverse genes.

**Supplementary Information:**

The online version contains supplementary material available at 10.1186/s12864-022-08653-y.

## Introduction

Physiological traits define how species live, the habitats they can exploit, health, and energy allocation between ecological interactions or reproduction [1–10]. To understand the molecular mechanisms and evolutionary forces altering physiological traits, it is critical to understand the genes involved. Quantifying mRNA expression provides information for both heritable and plastic responses [11–17], offering insight where genome wide association studies (GWAS) or similar approaches may be less informative due to context dependent genetic architecture [19, 20]. Studying mRNA expression is also likely to provide a greater mechanistic understanding of complex traits in comparison to genome wide nucleotide variation because mRNAs are more often defined genes associated with biochemical or physiological pathways (*e.g.,* through Kyoto Encyclopedia of Genes and Genomes [KEGG] or Gene Ontology [GO] terms or molecular investigation). Overall, an improved mechanistic understanding of traits enables us to parse redundancy across biological organization levels and reveal the evolutionary processes and genetic architectures contributing to phenotypic variation.

**Fig. 1:**
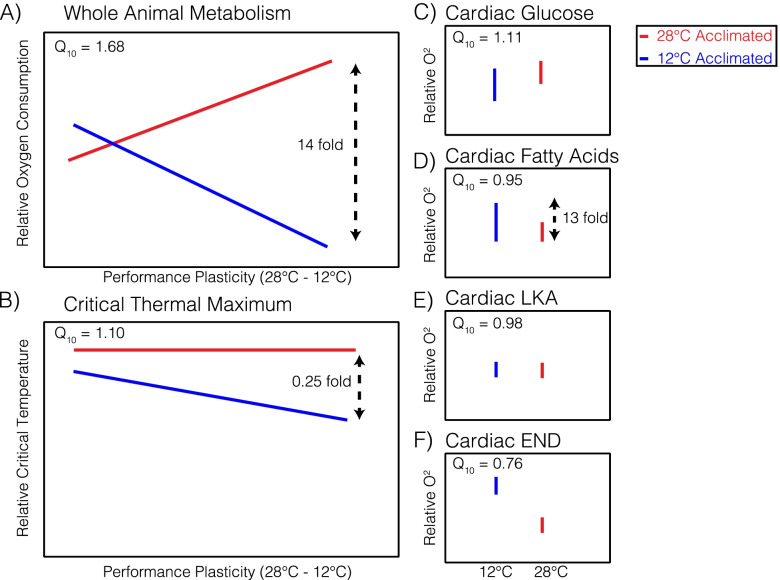
Metabolic and thermal tolerance trait variation among individual *Fundulus heteroclitus*. Relative physiological trait performance at 12 °C (blue) and 28 °C (red) acclimation and assay temperatures for **A)**Whole animal metabolic rate *versus* thermal plasticity between acclimation temperatures, **B** critical thermal maximum *versus* thermal plasticity between acclimation temperatures, and **C-F** cardiac metabolism under 12 °C and 28 °C acclimation and assay temperatures with substrates **C** glucose, **D** fatty acids, **E** lactate ketones and ethanol (LKA), and **F** endogenous (no substrate). Previously, difference between individuals when acclimated and assayed at 12 °C (blue) and 28 °C (red) revealing up to 14-fold variance in mass corrected whole animal metabolism (for the most plastic individual), 0.25-fold variance in critical thermal maximum, and 13-fold variance in fatty acid cardiac metabolism (the most variable substrate). Adapted from Drown et. al. 2021. [18]

Here, we used mRNA expression patterns to identify molecular mechanisms underlying physiological traits in the small saltmarsh teleost fish, *Fundulus heteroclitus*. Thermal physiology is often studied in *F. heteroclitus* because they are highly plastic and have adapted to live in the highly variable temperate coastal salt marshes where temperature fluctuates 15 °C on daily and seasonal timescales [18, 21–29]. Yet, few studies have examined the molecular and genetic basis of physiological trait variation related to thermal responses in this species beyond specific gene expression (*e.g.,* heat shock protein expression, [26, 30]), limiting our understanding of physiological response to temperature, which is likely to include 10 s or 100 s of expressed genes and may differ in response to variable acclimation conditions. To better understand thermal response molecular mechanisms in *F. heteroclitus*, we used individuals captured from three wild populations less than 15 km apart with little overall genetic distance among them [31–33] to examine physiological trait variation and then related physiological trait variation to heart and brain mRNA expression under 12 °C and 28 °C acclimation conditions (Fig. 1, [18, 34]). Traits included whole animal metabolic rate (WAM), critical thermal maximum (CT_max_), and four substrate specific cardiac metabolic rates (CaM substrates: glucose [Glu], fatty acids [FA], lactate + ketones + ethanol [LKA], and endogenous [END]) [18]. Notably, there were few differences among the three populations in the six physiological traits measured. This allowed us to address two hypotheses: 1) genetically similar populations with little divergence in physiological traits will have a shared mRNA expression response to thermal acclimation, and 2) physiological traits will be related to temperature and tissue specific mRNA expression, with variance among traits in the mRNAs that explain trait variation.

Using mRNA expression in combination with physiological trait variation we found co-variation between physiological traits and heart and brain mRNA expression among 86 individuals across the three populations. Among individuals, we previously found mass corrected physiological traits to be highly variable with up to 14-fold difference (range spread) in metabolic rate (mass corrected), 0.25-fold difference in CT_max_ (mass corrected), and 13-fold difference in fatty-acid CaM (heart mass corrected, the most variable substrate) [18] (Fig. 1, [18, 34]). This degree of trait variance is comparable to that found in metabolic rates [35] among 77 fish species spanning thousands of kilometers (15–20° latitude, ~ 2000 km (Fig. S1, data from [35]), and for thermal tolerance among polar and temperate crabs [36]. This large physiological trait variation among individuals within a species provides a powerful approach to determine the relationship between mRNA expression and these heritable fitness related traits [16, 37–41]. We show that among these individuals, acclimation induced differential expression of 362 heart mRNAs and 528 brain mRNAs across all three populations, yet few differentially expressed mRNAs (one or less) were shared across all three populations. Within each acclimation temperature, co-expressed mRNA modules were significantly associated with WAM, CT_max_, and CaM. Using KEGG and GO enrichment, we identify biologically relevant networks among co-expressed mRNA modules that explain these traits. These data link a simpler molecular phenotype (mRNA expression) to complex trait variation to enhance our understanding of biological pathways that affect these traits and may be important for evolutionary adaptation.

## Results

### Differential expression analysis

**Fig. 2:**
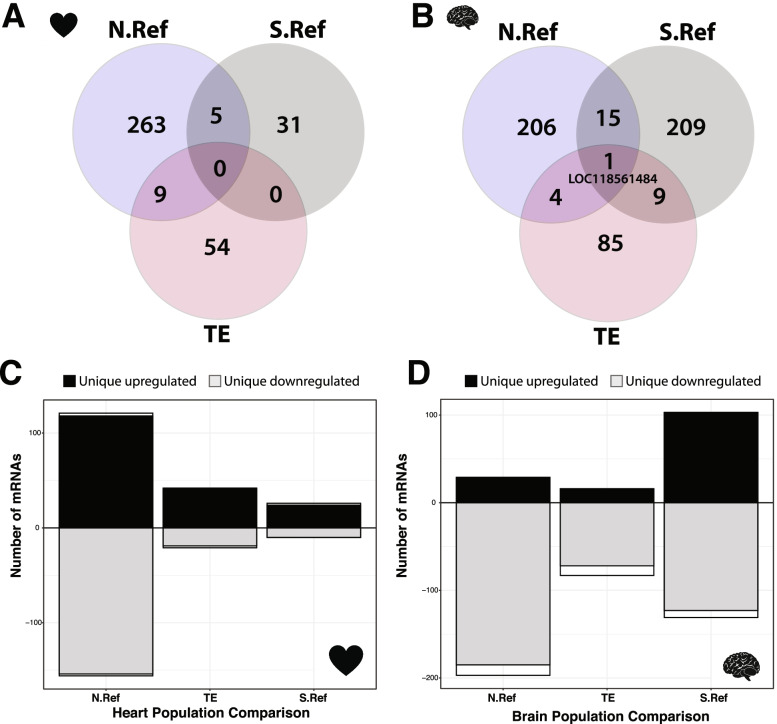
Population and tissue specific transcriptomic response to acclimation temperature. Number of differentially expressed mRNAs (DEGs) within each population between 12 °C and 28 °C acclimated hearts (**A **and** C**) and 12 °C and 28 °C acclimated brains (**B **and** D**). For both heart and brain, populations had many unique DEGs (**C **and** D**, upregulated = black, downregulated = grey) that were differentially expressed between acclimation temperatures and few shared DEGs (**C **and** D**, shared = white), with only 1 DEG shared among all three populations for brain (LOC118561484 in brains). Population and number of DEGs are not independent, Chi-Squared test, heart *p* = 1.64 × 10^–10^, brain *p* = 2 × 10^–16^

Sequencing statistics and sample sizes are summarized in Table 1. Differential expression patterns among populations and acclimation temperatures were identified using DESeq2 [42]. First, to examine population and temperature specific expression we used model design: (~ Population + Acclimation-Temperature + Population*Acclimation-Temperature). This analysis revealed significant Population*Acclimation-Temperature interactions, suggesting acclimation temperature specific mRNA expression patterns among populations. Because of the significant interactions, we analyzed individuals acclimated to 12 °C or 28 °C separately with model design: ~ Population to identify differentially expressed mRNAs among populations within an acclimation temperature. Similar to other species, there were significant differentially expressed mRNAs between acclimation temperatures, reflecting changes in response to environmental temperature ([14, 43, 44], Table S1). Across all 3 populations, hearts had 362 mRNAs (3.5% of total) that were significantly different between the two acclimation temperatures (FDR < 0.05) with an equal number of up and down regulated mRNAs for 12 °C *versus* 28 °C (180 up and 182 down). For brains, 528 mRNAs (4.8% of total) were significantly differently expressed between the two acclimation temperatures (FDR < 0.05) with ~ 2.5-fold more down regulated at 28 °C relative to 12 °C (148 up and 380 down). While all three populations showed acclimation effects for heart and brain mRNAs, the affected mRNAs were not shared among all populations (Fig. 2, Table S1). Additionally, acclimation significant mRNAs were unique to either heart or brain with no shared (0 mRNAs) acclimation response between tissues. This reflected different expression patterns between tissues, previously identified with PCA analysis (Fig. S2).

**Table 1:** Sequencing statistics. Sequencing statistics and sample size distribution among tissues, acclimation temperatures, and populations

**Heart Tissue**	**Sequencing Statistics**	**Acclimation Temperature**	**Population**	**Sample Size**
Total N	41	12 °C	N.Ref	4
Total mRNAs	10,535		TE	6
Average reads per mRNA	8,224.70		S.Ref	9
Minimum reads per individual	1.5 million	28 °C	N.Ref	4
Average reads per individual	2.17 million		TE	8
			S.Ref	10
			**Total**	41
**Brain Tissue**	**Sequencing Statistics**	**Acclimation Temperature**	**Population**	**Sample Size**
Total N	45	12 °C	N.Ref	9
Total mRNAs	10,932		TE	11
Average reads per mRNA	6578.5		S.Ref	8
Minimum reads per individual	1 million	28 °C	N.Ref	5
Average reads per individual	1.74 million		TE	6
			S.Ref	6
			**Total**	45

**Fig. 3:**
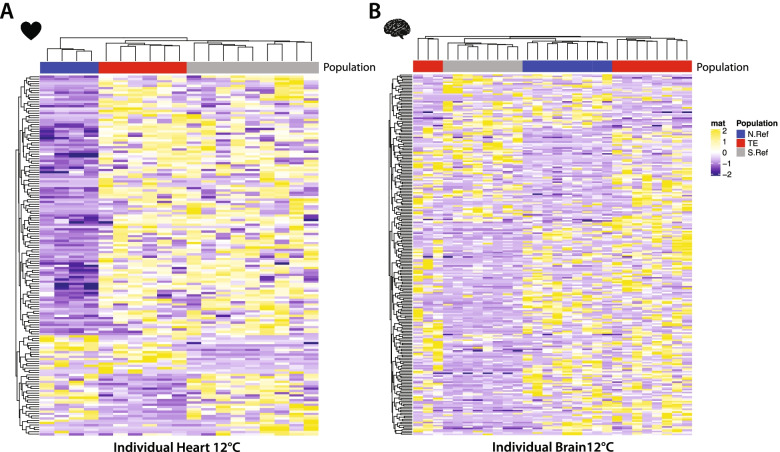
Temperature specific differential mRNA expression among populations. **A** Differential expression for 158 heart mRNAs and **B** 242 brain mRNAs between any two populations at 12 °C. In hearts, 43.0% of mRNAs (68/158) are shared among any two population comparisons. In brains, 32.2% of mRNAs (78/242) are shared among any two population comparisons

At each acclimation temperature, populations also had significant expression differences (Fig. 3, Table S2). Hearts at 12 °C and 28 °C had 158 or 153 differentially expressed mRNAs among populations, respectively (Table S2). These represent 1.50% or 1.45% of all expressed heart mRNAs at 12 °C and 28 °C, respectively; brains had 242 or 330 differentially expressed mRNAs among populations at 12 °C and 28 °C, respectively. These represent 2.21% or 3.02% of all expressed brain mRNAs at 12 °C and 28 °C, respectively. None of the population effects were significant across all three populations (Fig. S4) for any acclimation temperature or tissue.

Importantly, there is an adaptive hypothesis: that the mRNAs in the anthropogenically warmed population, TE, are uniquely different, where TE is significantly different from both northern and southern reference populations with no significant differences between the references [14, 45]. For heart mRNAs at 12 °C there are 10 mRNAs (6.33% of significant mRNAs), and at 28 °C there are 3 mRNAs (1.96%) where the TE population is uniquely different from both references (Fig S4, Table S2). For brain mRNAs at 12 °C there are 11 mRNAs (4.55% of all significant mRNAs), and at 28 °C there are 27 mRNAs (8.18%) where the TE population is uniquely different from both reference populations. While the overall frequency of differentially expressed genes is small (1.45% to 3.02% *vs.* total 10 k mRNAs), the pattern where the TE population is different from both northern and southern reference populations but the two reference populations are not different aligns with an adaptative hypothesis.

### Variation in mRNA expression

In addition to differential expression analysis, we were interested in the degree of mRNA expression variance. Previously, we found that variation in WAM, CT_max_, and substrate specific CaM was greater at 12 °C than at 28 °C. Here we found that both heart and brain tissue had greater average coefficient of variation (CV, standard deviation/mean*100) across all mRNAs at 12 °C than at 28 °C (T-test, heart *p* = 9.587e-05, brain *p* = 0.02014), similar to our findings of greater variation in physiological traits measured at 12 °C.

### Weighted gene co-expression network analysis

We used weighted gene co-expression network analysis (WGCNA, [46]) to detect co-expressed mRNA clusters. WGCNA approaches group mRNAs with similar expression patterns into independent modules. Expression patterns for all mRNAs within a module were then summarized into principal components called module eigengenes (MEs, Table 2 for heart mRNAs and Table 4 for brain mRNAs), and these MEs were correlated to each of the six physiological traits (Table 3 shows significant heart ME trait correlations, and Table 5 shows significant brain ME trait correlations). Each ME has a “hub-MM”, the mRNA with the highest correlation to the ME, with MM being the correlation coefficient (Tables 2 and 4).

**Table 2:** Heart Significant Modules

Module	KEGG Pathway	Module Size	Hub MM	MM	Positive MM	KEGG Terms in Pathway
ME1_heart	MAPK signaling pathway*	194	SAC3D1	0.51	74.70%	K04459
						K20216
ME2_heart	Endocytosis*	147	NCKAP5L	0.52	31.30%	K12471
ME3_heart	FoxO signaling pathway*	281	CLMPB	0.53	23.80%	K11411
ME4_heart	RNA degradation	168	TRAFD1	0.49	32.10%	K03681
ME5_heart	Metabolic pathways	336	LOC105935504	0.39	30.90%	K00166
						K00232
						K03844
						K05546
ME6_heart	NA	554	WNT5B	0.65	34.10%	NA
ME7_heart	NA	454	MOSPD2	0.57	30.60%	NA
ME8_heart	Oxidative phosphorylation*	142	TXLNA	0.45	32.40%	K0393
ME9_heart	Ribosome*	90	YIF1B	0.54	66.70%	K02899

Columns include: Module = identifier for module eigengene (ME, first principal component of module), KEGG Pathway = top KEGG pathway determined by number of KEGG terms, Module Size = number of mRNAs in the module, Hub MM = mRNA with highest correlation with module eigengene, MM = correlation of hub mRNA with module eigengene, Positive MM = proportion of mRNAs with positive MM in the module, KEGG Terms in Pathway = enriched KEGG terms in the listed KEGG pathway.

*Indicates modules where more than one KEGG Pathway had the same number of enriched KEGG terms, in which case the most informative KEGG pathway was selected

**Table 3:** Heart Significant Module Trait Correlations

Trait	Module	Correl coef	FDR *P*-value	Hub GS	GS	Positive GS
CTMax 12 °C	ME1_heart	0.49	2.10E-02	GPM6AA	-0.51	54.60%
CTMax 28 °C	ME2_heart	-0.53	9.50E-03	NCKAP5L	-0.50	35.30%
FA 12 °C	ME3_heart	0.50	1.02E-02	PDCD4A	0.55	65.80%
FA 12 °C	ME4_heart	0.53	4.82E-03	RSRC1	-0.58	13.10%
FA 12 °C	ME5_heart	0.55	4.82E-03	RPL5A	0.62	81.80%
heart mass 12 °C	ME6_heart	-0.57	1.80E-03	WRAP53	0.72	48.20%
LKA 12 °C	ME6_heart	-0.65	3.39E-05	LOC118563371	0.80	46.90%
LKA 12 °C	ME7_heart	-0.56	1.29E-03	LOC105934375	0.80	44.70%
WAM 12 °C	ME8_heart	-0.56	1.20E-03	LOC118563115	0.70	36.60%
WAM 12 °C	ME9_heart	-0.55	1.20E-03	LRRC58B	-0.56	10.00%
WAM 12 °C	ME4_heart	-0.54	1.74E-03	LOC118563570	0.61	65.50%
WAM 12 °C	ME5_heart	-0.56	1.20E-03	HPRT1L	0.66	40.50%
	AVERAGE	0.55				

Significant heart ME correlations with FDR *p* < 0.05. Columns include: Trait = traits significantly correlated with a given ME (critical thermal maximum: CT_max_, whole animal metabolic rate: WAM, cardiac metabolic rate: CaM with substrates fatty acids = FA, lactate + ketones + ethanol = LKA), Module = identifier for module eigengene (ME, first principal component of module), Correl coef = Pearson’s signed correlation coefficient for trait and ME, FDR *P*-value = multiple test corrected *p*-value for trait *versus* module correlation, Hub GS = mRNA with highest gene significance for the trait in the module, GS = gene significance, correlation between top module mRNA and trait, Positive GS = proportion of mRNAs in the module that are positively correlated with trait

**Table 4:** Brain Significant Modules

Module	KEGG Pathway	Module Size	Hub MM	MM	Positive MM	KEGG Terms in Pathway
ME1_brain	Metabolic pathways	393	IDH3B	0.47	26.40%	K00323
						K01443
						K10106
						K19006
ME2_brain	Tight junction	198	LOC105921153	0.52	28.20%	K07198
						K08018
						K08020
ME3_brain	Lysosome	342	LRCH3	0.58	25.10%	K01134
						K08568
ME4_brain	RNA degradation*	142	SI:CH211-244C8.4	0.36	21.10%	K03681

Columns include: Module = identifier for module eigengene (ME, first principal component of module), KEGG Pathway = top KEGG pathway determined by number of KEGG terms, Module Size = number of mRNAs in the module, Hub MM = mRNA with highest correlation with module eigengene, MM = correlation of hub mRNA with module eigengene, Positive MM = proportion of mRNAs with positive MM in the module, KEGG Terms in Pathway = enriched KEGG terms in the listed KEGG pathway

*Indicates modules where more than one KEGG Pathway had the same number of enriched KEGG terms, in which case the most informative KEGG pathway was selected

MEs were correlated to the body mass residuals for the six traits (WAM, CTmax, and the four substrate specific CaM). These analyses were done across all three populations because populations did not have any significant differences among traits. Each of the ME-trait correlations had a “hub-GS” – the mRNA in the module with the highest correlation to the trait, with GS (gene specific) being the correlation coefficient for this single mRNA. Both heart and brain WGCNA analysis used a minimum module size of 30 mRNAs per module and combined modules with correlation > 75% (see methods). To verify that trait *versus* ME correlations were not driven by spurious outliers, we used a jack-knife approach to subsample 90% of individuals and repeat ME-trait correlations 100 times. Correlations that were significant in at least 70 out of 100 repeated correlations in the same direction (positive or negative correlation coefficient) were retained for further analysis (see methods).

#### Heart WGCNA

**Fig. 4:**
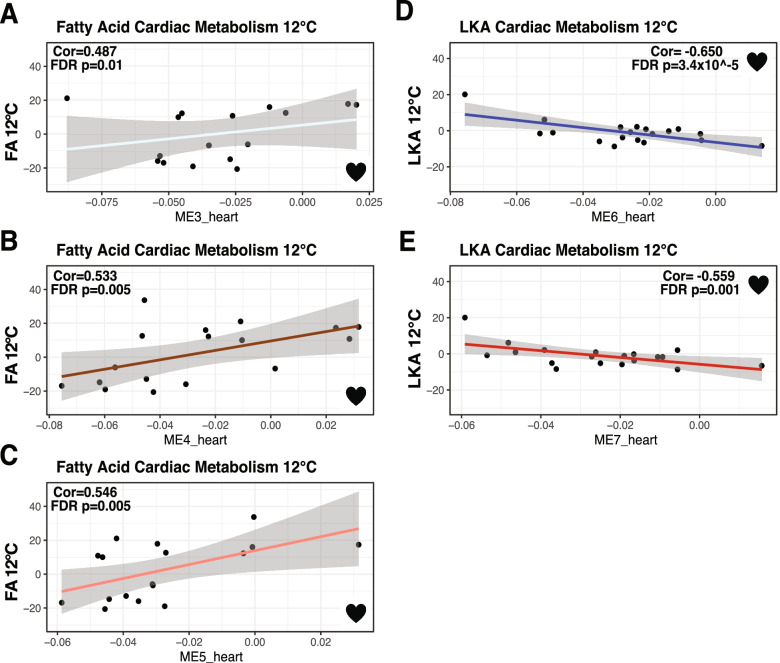
Significant cardiac metabolism-heart module correlations from weighted gene co-expression network analysis. Significant correlation of fatty acid cardiac metabolic rate at 12 °C (*N* = 16) with ME3_heart (**A**), ME4_heart (**B**), and ME5_heart (**C**). Significant correlation of lactate, ketone, and alcohol (LKA) cardiac metabolic rate at 12 °C (*N* = 19) with ME6_heart (**D**) and ME7_heart (**E**). Pearson correlation coefficients (Cor) and FDR *p*-values are displayed for each significant correlation

**Fig. 5:**
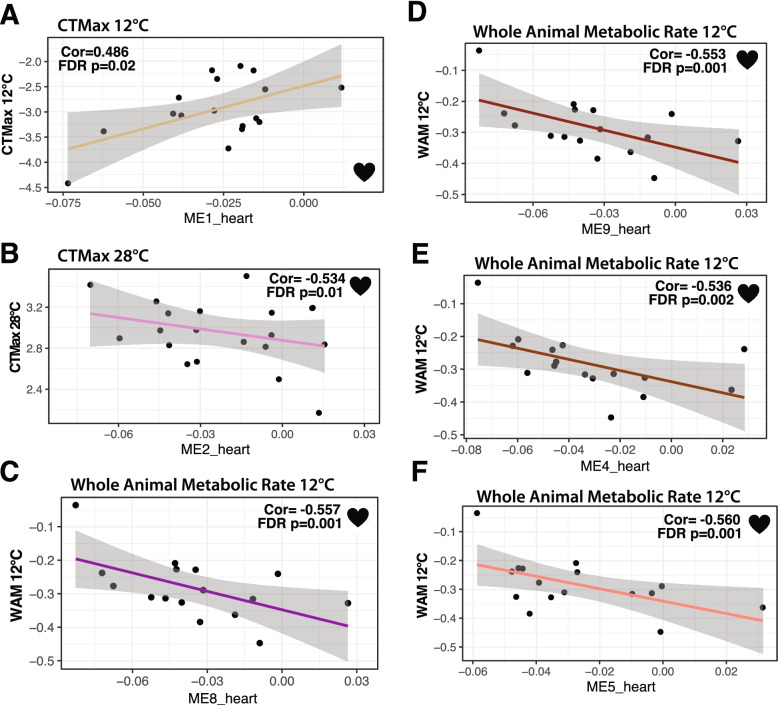
Significant whole animal trait-heart module correlations from weighted gene co-expression network analysis. Significant correlation of critical thermal maximum at 12 °C (*N* = 17) with ME1_heart (**A**), critical thermal maximum at 28 °C (*N* = 19) with ME2_heart (**B**), whole animal metabolic rate at 12 °C (*N* = 16) with ME8_heart (**C**), ME9_heart (**D**) ME4_heart (**E**), and ME5_heart (**F**). Pearson correlation coefficients (Cor) and FDR *p*-values are displayed for each significant correlation

For heart mRNAs, we found 39 co-expression modules with 90 to 554 mRNAs in each module (Table 2), and these heart MEs (first principal component of co-expressed mRNAs) were correlated to six physiological traits at each acclimation temperature. There were 12 significant ME-trait correlations: 9 heart MEs with 5 temperature specific traits (FDR < 0.05, Table 3, Figs. 3 and 4). Traits correlated with at least one of these 9 heart MEs included: at 12 °C WAM, CT_max_, FA CaM, heart mass, LKA CaM, and at 28 °C, CTmax (Table 3, Figs. 4, 5). Two of these modules (ME4_heart, ME5_heart) were correlated with both WAM at 12 °C and FA CaM at 12 °C, and one module (ME6_heart) was correlated with both LKA CaM at 12 °C and heart mass at 12 °C. WAM at 12 °C had the most significant ME correlations (4 total), followed by FA CaM at 12 °C (3 total) and LKA CaM at 12 °C (2 total). The other three traits were each correlated with a single module (Table 3). On average, a single heart module explained 55% of variance for one trait with a minimum of 48.5% (ME1_heart with CT_max_ 12 °C) and a maximum of 65% (ME6_heart with LKA 12 °C).

For traits that were significantly correlated with more than one ME, a multiple correlation coefficient was calculated. For WAM at 12 °C, the four significant MEs together had a multiple correlation coefficient of 82%, the three significant MEs for FA CaM at 12 °C had a multiple correlation coefficient of 79.5%, and the two significant MEs for LKA CaM at 12 °C had a multiple correlation coefficient of 75.5%. All modules correlated with FA CaM at 12 °C and CT_max_ at 12 °C had positive correlation coefficients while all other significant trait *versus* ME correlations in hearts had negative correlation coefficients.

#### Brain WGCNA

**Fig. 6:**
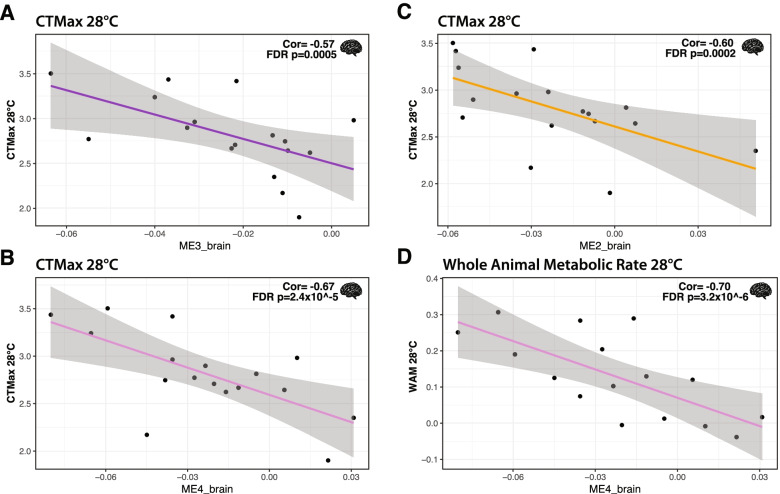
Significant whole animal trait-brain module correlations from weighted gene co-expression network analysis. Significant correlation of critical thermal maximum at 28 °C (*N* = 17) with ME3_brain (**A**), ME4_brain (**B**), and ME2_brain (**C**). Significant correlation of whole animal metabolic rate at 28 °C (*N* = 16) with ME4_brain (**D**). Pearson correlation coefficients (Cor) and FDR *p*-values are displayed for each significant correlation

For brain mRNAs, we found 42 co-expressed modules with 142 to 393 mRNAs per module (Table 4). There were 6 significant ME-trait correlations (FDR < 0.05) that included 4 unique modules and 4 temperature specific traits (Table 5, Fig. 6): at 12 °C, body mass and at 28 °C, CT_max_, WAM, and body mass. The trait with the most significant correlations was CT_max_ at 28 °C (3 significant ME’s), and two of these were also significant with body mass at 28 °C (ME3_brain) or WAM at 28 °C (ME4_brain). On average, the correlation coefficient for a brain ME was 62% with a minimum of 56.8% (ME3_brain with CT_max_ 28 °C) and a maximum of 70.2% (ME4_brain with WAM 28 °C). For CT_max_ at 28 °C, which was correlated with three MEs, the multiple correlation coefficient was 71.7%. All correlations between traits and brain MEs were negative except for body mass at 28 °C.

**Table 5:** Brain Significant Module Trait Correlations

Trait	Module	Correl coef	FDR *P*-value	Hub GS	GS	Positive GS
body mass 12 °C	ME1_brain	-0.60	5.75E-04	MAP3K5	0.61	44.80%
body mass 28 °C	ME3_brain	0.58	1.46E-03	LOC105919139	0.63	68.70%
CT_max_ 28 °C	ME2_brain	-0.60	2.01E-04	CIAO2B	0.36	44.90%
CT_max_ 28 °C	ME3_brain	-0.57	4.83E-04	RAD17	0.64	83.90%
CT_max_ 28 °C	ME4_brain	-0.67	2.38E-05	APOC1	-0.65	48.60%
WAM 28 °C	ME4_brain	-0.70	3.15E-06	APOC1	-0.68	50.00%
	AVERAGE	0.62				

Significant brain *versus* ME correlations with FDR *p* < 0.05. Columns include: Trait = traits significantly correlated with a given ME (critical thermal maximum: CT_max_, whole animal metabolic rate: WAM), Module = identifier for module eigengene (ME, first principal component of module), Correl coef = Pearson’s signed correlation coefficient for trait and ME, FDR P-value = multiple test corrected *p*-value for trait *versus* module correlation, Hub GS = mRNA with highest gene significance for the trait in the module, GS = gene significance, correlation between top module mRNA and trait, Positive GS = proportion of mRNAs in the module that are positively correlated with trait

### KEGG and GO enrichment

#### Critical thermal maximum enriched terms

MEs were tested for KEGG and GO term enrichment using the complete set of tissue specific mRNAs as the gene universal or reference set (10,535 for heart, 10,932 for brain; Tables S3 and S4). For CT_max_, all 5 MEs were significantly enriched for KEGG pathways (ME1_heart, ME2_heart, ME2_brain, ME3_brain, and ME4_brain) and included MAPK signaling, mTOR signaling, glyoxylate and dicarboxylate metabolism, insulin signaling pathway, glutathione metabolism, metabolic pathways, carbon metabolism, and tryptophan metabolism. Enriched GO terms included regulation of organ growth and cellular stress response in heart and AMP-activated protein kinase (AMPK) activity in brain. ME for CT_max_ at 28 °C contained substantial overlap in enriched KEGG pathways related to metabolism with 5 out of 8 terms enriched in both heart and brain modules. So, although there were different mRNAs in heart and brain modules correlated with CT_max_ at 28 °C, the KEGG terms related to metabolism were shared (5 out of 8), suggesting that different mRNAs in heart and brain belonged to similar pathways impacting CT_max_ at 28 °C.

#### Metabolic rate enriched terms

Modules significantly correlated with WAM were enriched for KEGG pathways including oxidative phosphorylation (heart only), glutathione metabolism (brain only), and metabolic pathways (both heart and brain). In addition, ME3_heart was correlated with FA CaM at 12 °C and enriched for KEGG terms including metabolic pathways, forkhead protein (FoxO) signaling, and metabolism of NADH derivatives (nicotinate and nicotinamide). One module, ME5_heart, was significantly correlated with WAM at 12 °C and FA CaM at 12 °C and contained several KEGG pathways directly related to fatty acid metabolism as well as known transcription factors like PPAR that impact metabolic homeostasis by controlling expression of many metabolism related genes [47]. Previously, partial correlation coefficients between FA CaM at 12 °C and WAM at 12 °C were negatively correlated [18], and similarly ME_5 had opposite correlation coefficients for these two traits (Table 3, Figs. 4, 5). This correlation between traits and their correlations to MEs also occurs for CT_max_ and WAM at 28 °C [18] and ME4_brain (enriched for glutathione metabolism and metabolic pathways). The fact that the same MEs are associated with traits that have significant partial correlations emphasizes the biological relevance of the MEs.

## Discussion

Understanding the genes that affect physiological performance is one of the more important research goals for human health [48–50] and evolutionary ecology, especially with global climate change [2, 6, 10, 51–53]. Here, to provide insight into the genes that affect physiological performance, we relate the mRNA variation with the variation in six physiological traits (Fig. 1, [18, 34]). This approach is strengthened by the large variation among *F. heteroclitus* individuals used in this study: the variation (range spread) is 14-fold for WAM, 13-fold for FA CaM, and 0.25-fold for CT_max_ [18] (Fig. 1). These physiological trait variations are larger than found among different species spanning temperate to tropical waters (Fig. S1). Additionally, five of six traits are not significantly different among the three populations (*F*-values < 2), with most trait variation found among individuals and not among populations [18]. This substantial interindividual variation in traits allowed us to examine interesting physiological patterns related to acclimation response and temperature performance. For example, individuals with low WAM at 12 °C tended to have high WAM at 28 °C and visa-versa; the substrates that supported CaM differed among individuals; and, acclimation response eliminated temperature effects such that Q_10_ (change in rate for every 10 °C increase in temperature) was approximately 1.0 for cardiac metabolic rates (nearly equal rate when measured at 12 °C and 28 °C, [18]). The large physiological variation among individuals, the difference in substrate use, and the strong acclimation responses provide a unique resource to begin to understand which mRNAs are associated with physiological performance and thus the genes responsible for health and response to environmental change.

### The role of mRNA expression in acclimation and evolution

For mRNA expression in both heart and brain tissues, we found significant interactions between acclimation and population effects: the expression of several hundred mRNAs differed between acclimation temperatures, but these were not shared among all three populations (Fig. 2). Previously (Fig. 1., [18]), in these same individuals, acclimation response to 12 °C and 28 °C affected all six physiological traits (WAM, CT_max_, and the four substrate specific CaM). For CT_max_, there was an expected enhancement: higher CT_max_ in individuals experiencing warmer environments. For metabolic rates (WAM and CaM), acclimation to 12 °C and 28 °C mitigated the effect of temperature [18]. Specifically, without physiological acclimation there is an expected ~ threefold increase in metabolic rates with the 16 °C increase in acclimation and assay temperature (*i.e.,* with a doubling for every 10 °C) [54]. Yet, WAM had only ~ 1.2-fold increase [43] from 12 °C to 28 °C, and CaM had no significant increase between temperatures [18]. Presented here, across all three populations, acclimation produced significant differential mRNA expression (FDR < 0.05) in hearts (362 mRNAs) and brains (528 mRNAs, (Table S1)). These mRNA expression changes associated with acclimation responses are similar to prior studies among ectotherms where transcriptomic response to temperature acclimation enhances thermal performance [43, 44, 55, 56]. For example, in three-spine stickleback (*Gasterosteus aculeatus*) and other fishes, metabolic enzyme expression and mitochondrial volume density increase in response to cold acclimation can compensate for reduced enzyme catalytic rate with decreased temperature [43, 57, 58]. Similarly, in eastern oysters (*Crassostrea virginica*) among a suite of environmental factors (temperature, pH, salinity, dissolved oxygen, etc.), temperature was the most important transcriptomic variation predictor with thermal stress increasing oxidative phosphorylation transcript expression [59]. Even Antarctic fish, which are adapted to extreme and invariable cold, show plasticity in metabolic transcripts with temperature acclimation that impacts whole animal performance [58, 60].

While quantitative gene expression changes are common with acclimation (affecting both mRNA and proteins [55, 61]), surprisingly, mRNA acclimation responses were different among populations—for heart mRNAs, no significant acclimation responses were shared among all three populations, and for brains only one mRNA was shared among the three populations. Further, 88–98% of significant acclimation responsive mRNAs are unique to each population. In contrast, the six physiological traits’ acclimation responses were not different among populations. All populations were subjected to a common environment for a long time (~ 1 year or nearly 30–50% of a fish’s expected life span) with acclimation to the 12 °C and 28C. Thus, the difference in acclimation mRNA response among populations was not due to short-term physiological effects and may be due to genetic polymorphisms driving acclimation responses but could also be due to irreversible developmental effects or trans-generational effects. Regardless of the genetic mechanisms responsible for the divergent mRNA acclimation responses among populations, these data suggest that multiple different mRNA expression patterns drive acclimation responses. This conclusion is similar to CaM measurements in Maine and Georgia populations, where the mRNAs that explain substrate specific metabolism varied among groups of individuals [16]. The observations, that plasticity in the six physiological traits between temperatures is similar among populations yet mRNA acclimation responses differ among populations, suggest that multiple redundant molecular mechanisms drive temperature compensation.

A single difference occurs among populations for the six physiological traits: endogenous CaM at 28 °C. Yet, populations had significant mRNA expression differences specific for each temperature, and none of the population significant mRNAs were shared at 12 °C or 28 °C (Fig. S4, Table S2). One pattern indicative of adaptation occurs where the anthropogenically warmed TE population is significantly different from both northern and southern reference populations (not heated by thermal effluent from nuclear power plant) but not different between these two references [14, 31, 45]. While we did not find this adaptive pattern for any of the physiological traits, a few mRNAs (2–8%) did show this pattern. Yet, this pattern is not unlikely given the number of differences among all populations. That is, it is probable that 2%-8% of mRNAs for either tissue or at either acclimation temperature would have this potentially adaptive pattern of mRNA expression. Thus, while some mRNA expression patterns are exclusive for the anthropogenically heated TE population relative to both references, an adaptive hypothesis is possible but not strongly supported by our mRNA data. Regardless, the differences here were for mRNA expression that can be affected by DNA polymorphisms or influenced by environment (*i.e.*, GxE). Thus, the difference among populations in mRNA expression are dependent on the thermal environment, and, if adaptive, suggest that the different genetic polymorphisms are responsible for adaptive divergence at different temperatures. This conclusion is similar to a comparison within and among species across a wider geographic range where adaptive divergence in mRNA expression was dependent on the thermal environment [14]. For the TE population, Dayan et al*.* [31]*,* concluded that there was adaptive divergence based on evolutionarily significant DNA polymorphisms. We would extend this to suggest that populations have evolved different mRNA expression patterns that are dependent on the thermal environment but that, nevertheless, produce similar physiological phenotypes. This is consistent with a redundant polygenic trait architecture, which may be expected for some complex traits that are affected by many physiologically processes [48]. While mRNA expression variation is largely attributed to DNA polymorphisms [62, 63], other possible mechanisms including epigenetic modification (*e.g.,* DNA methylation) are also known to impact mRNA expression and may impact the physiological trait variation we have measured [64–66].

### Biological relevance of co-expressed mRNAs

This study’s focus is to identify differential mRNA expression responsible for the large physiological trait variation previously characterized [18]. We examine co-variation among mRNAs and relate this to the six physiological traits using a weighted gene co-expression network analysis (WGCNA) [46]. WGCNA identified co-expressed mRNA modules, MEs, highly correlated with WAM, CT_max_, FA CaM, LKA CaM, and body and heart mass, depending on the acclimation temperature (Figs, 4, 5, and 6). The average ME-trait correlation was 0.55 for heart and 0.62 for brains (Tables 3 and 5). These MEs, containing 90–554 mRNAs each, contained few (0–10) mRNAs with significant expression differences among populations, suggesting that the variation among individuals, and not differences among populations, is most important. This is in agreement with five of the six physiological traits, where most the variation is within and not between populations (exception of CaM END at 12 °C). WGCNA has been previously used to identify mRNA expression networks important for various pathologies including cardiovascular disease [67, 68], cancers [69–73], and diabetes [68, 74], among others. In non-human organisms, WGCNA has been used to characterize response to the environment, including heat stress in turbot [75], carotenoid metabolism in apricot fruit [76], and disease response in corals [77]. Although few studies, to our knowledge, have validated correlations using jack-knife subsampling to ensure that the correlations were consistent among most individuals and not driven by a few outliers as we did, these studies similarly identified potentially meaningful correlations between traits and co-expressed mRNAs.

Our jack-knife subsampling indicates that the high correlations between traits and co-expressed mRNAs are found among most individuals and not driven by a few outliers. More importantly, in this study, the correlation patterns between MEs and physiological traits are similar to the correlations among physiological traits. For example, at 12 °C, FA CaM and WAM were negatively correlated [18], and similarly, ME5_heart was significantly correlated with opposite signs with these two traits (*i.e.,* positively correlated with FA CaM but negatively correlated with WAM, Table 3, Figs. 4, 5). Additionally, MEs correlated to WAM and CaM were enriched in KEGG metabolic pathways and GO terms related to metabolism. These data indicate that modules represent independent, biologically important mRNA networks.

The biological importance of co-expressed mRNA networks is also supported by their relation to metabolic processes. Eleven of the 13 significant heart or brain MEs were significantly enriched for at least one KEGG term, and 6 were significantly enriched for at least one GO term. KEGG terms mapped to biologically relevant KEGG pathways including metabolic pathways, mechanistic target of rapamycin (mTOR) signaling, mitogen activated protein kinase (MAPK) signaling, insulin signaling, and metabolism and biosynthesis of various macromolecules including glycogen, NADH precursors, amino sugars, and fatty acids (Table S3, S4). Importantly, 8 out of 11 modules with significantly enriched KEGG terms mapped to at least one metabolism related KEGG pathway. Among these KEGG terms are an abundance of signaling pathways that are known to affect physiological systems (see below). Yet, we also found several enriched KEGG pathways and GO terms that were uniquely enriched in only one or few modules and seemingly unrelated to the correlated trait(s) (*e.g.,* cellular senescence). This could indicate a limited understanding of the complexity and interconnectedness among biological pathways and how different pathways affect a diversity of traits, mRNAs that are minimally annotated and missing relevant pathway involvement, or mRNA expression that impacts biological processes that indirectly impact the traits we have measured. The concept that there is a limited understanding of the interactions among pathways is supported by earlier mitochondrial respiration studies, specifically concerning the oxidative phosphorylation pathway (OxPhos) [78]. Among selectively important nuclear genes affecting OxPhos, none of the genes were among the 97 proteins in the OxPhos pathway; instead, they were in diverse pathways, some of which made sense (*e.g.*, ADP transport- where ADP is a substrate for OxPhos) [78]. Thus, the few MEs associated with unexpected pathways may indicate a complexity in physiological traits where many pathways and the genes in these pathways affect trait variation.

Previously, data from our laboratory demonstrated that natural variation in substrate specific cardiac metabolism in *F. heteroclitus* could be explained by cardiac mRNA expression using microarray data [16]. Similar to the WGCNA approach presented here, the first principal component of mRNA expression from different metabolic pathways (oxidative phosphorylation, TCA cycle, glycolysis) explained substrate specific CaM among individuals with different mRNA pathways explaining substrate specific metabolisms in different individuals. Here, we found that mRNA expression explained a similar proportion of substrate specific CaM as previously reported (~ 80%) using three MEs to explain a single trait (FA CaM at 12 °C).

Few, if any, studies have examined the correlation between co-expressed mRNA and CT_max_ (although see [79]). Our analyses found that 341 heart mRNAs in two co-expressed modules and 682 brain mRNAs in three co-expressed modules were associated with CT_max_ at 12 °C or 28 °C, with different MEs at each temperature. Furthermore, heart and brain significant MEs for CT_max_ at 28 °C share enriched KEGG pathways, yet do not share any mRNAs, suggesting that different mRNAs affect a common set of pathways that impact CTmax. These data suggest that CTmax is polygenic and relies on different mRNAs in different tissues at different temperatures. There is prior evidence suggesting that CT_max_ is polygenic: a GBS study covering ~ 0.1% of the genome found up to 47 single nucleotide polymorphisms (SNPs) that explained 43.4% of variation in CT_max_ among *F. heteroclitus* individuals collected from Georgia, New Jersey, and New Hampshire, USA [28]. Here, a greater proportion of CT_max_ was explained with mRNA expression, up to 71.7% with 3 brain MEs. This increase in explained CT_max_ variance by mRNA expression is likely due to the combined heritable and physiologically inducible nature of mRNA expression. Few (0–10) of the mRNAs in MEs were differentially expressed between 12 °C and 28 °C, and thus MEs that explained CT_max_ variation within each of acclimation temperatures are not due to acclimation effects on mRNA expression. Instead, the CT_max_ variation within each acclimation temperature appears to be due to individual variation in mRNA expression, which may be explained by nucleotide variation driving differential expression.

Whole animal metabolism, WAM, is a fundamental physiological process that defines how animals live, niche space, evolutionary transition, and the human condition [2, 4, 6, 7, 80]. There is significant literature investigating metabolic rate variation (*e.g.* [41, 81, 82]*,*); however, the relationship between metabolic rate and mRNA expression remains poorly understood. This may be due to the complex nature of whole animal metabolism, which is a sum of tissue specific metabolic demands and a balance between growth, maintenance, and energy storage. Yet, we find 82% of 12 °C WAM variation related to four heart MEs with 736 mRNAs and 50% of 28 °C WAM variation related to one brain ME with 142 mRNAs. These data indicate that a large proportion of WAM can be explained by mRNAs within a common pathway impacting cardiac metabolic processes and thus provides insight into the physiological relationship between cardiorespiratory performance and overall metabolism [83–86].

These WGCNA analyses suggest that many mRNAs in several biochemical pathways define the physiological state among individuals. Yet, the careful reader will note two substantial complexities: 1) heart MEs explain the variation in many physiological traits at 12 °C but few at 28 °C, and brain MEs explain the variation in many physiological traits at 28 °C but few at 12 °C and 2) mRNAs within MEs are enriched for many diverse and unexpected pathways as discussed above.

The difference between tissue specific MEs and their association with physiological traits is related to CaM, WAM, and CT_max_ having higher inter-individual variation at 12 °C than 28 °C, and similarly there is greater mRNA expression variation at 12 °C than at 28 °C. Thus, the more frequent explanation of physiological traits by 12 °C mRNA expression may simply result from greater statistical power due to the greater variance in both physiological traits and mRNA. Yet, in brains, mRNAs explain WAM and CT_max_ at 28 °C. While we can only speculate, these data suggest that at the higher temperature brain mRNA expression is more important than cardiac mRNA expression. There is evidence that acclimatory response to temperature in brain is greater than in hearts (more acclimatory mRNAs and greater decreased mitochondrial function in brain when compared to heart tissue [24]). This is similar to our data: brains at 28 °C have more acclimation responsive mRNAs than hearts and more population divergence than brains at 12 °C or hearts at either temperature. Together these data suggest that the variation in WAM and CTmax at 28 °C are more dependent on brain specific expression.

Many MEs are associated with KEGG pathways that impinge on metabolic processes. Physiological processes are within 9 heart MEs and 4 brain MEs (Tables 2 and 4), and these MEs each contain 90–554 mRNAs. Each of these MEs is significantly enriched for multiple KEGG and GO pathways (Tables S3 and S4). Surprisingly MEs that explain trait variance do not mainly include genes involved in primary metabolic pathways (*e.g.*, glycolysis, TCA cycle, or oxidative phosphorylation) but instead are enriched for several signaling pathways including MAPK, mTOR, AMPK, PPAR, and FoxO. These signaling pathways are known to impact metabolic and thermal tolerance among ectotherms. For example, MAPK has been linked to adaptive cold tolerance [55, 87] and lipid metabolism [88]. Additionally, mTOR is involved with energy homeostasis, has been linked to growth and longevity, and may be sensitive to temperature variation [89–91]. AMPK induces cellular ATP production in mammals and is important for thermal stress response in ectotherms [92–94]. Furthermore, AMPK phosphorylation in Coho salmon and rainbow trout hearts has been correlated with exposure above optimum temperatures [95], suggesting a role of AMPK in fish thermal response. FoxO proteins, especially FoxO1, are involved in energy homeostasis and may aid in the switch from carbohydrate (glycolytic) to fatty acid metabolites [96]. Therefore, although these pathways may not be the “usual suspects”, their role in ectotherm metabolism and thermal tolerance is well documented, and we suggest that signaling pathways play an important role in explaining the trait variation examined here. In addition to these important signaling pathways, several modules are enriched for pathways not typically thought to be directly involved in metabolism or thermal tolerance. Similar to nuclear genes that impact *Fundulus* mitochondrial respiration [78], these data suggest that many metabolically distant genes affect physiological variation. This is important because too often publications have “just so stories” (*e.g.*, [97, 98]) that only focus on a few preconceived genes to explain functional physiological variation [99]. While it is understandable to highlight a prior expectation, doing so limits our understanding of how genotypes effect phenotypes.

## Conclusion

In summary, these data address two hypothesis, first, that genetically similar populations with little divergence in physiological traits would have a shared mRNA expression response to thermal acclimation. Here, we found that mRNAs important for acclimation are population specific and that divergence among geographically close populations did not include acclimation responsive mRNAs. This suggests that even genetically similar populations have distinct thermal response molecular mechanisms. Our second hypothesis was that physiological traits would be related to temperature and tissue specific mRNA expression, with variance among traits in the mRNAs that explain trait variation. We found this to be true, with tissue specific mRNA expression associated with physiological traits dependent on the thermal environment. We highlight that biologically important mRNA networks are related to 48–82% of variation in whole animal metabolism, thermal tolerance, or substrate specific cardiac metabolism and are different at different thermal environments. This suggests that mRNA variation among individuals within and among populations is important for explaining complex trait variation and, surprisingly, that while similar pathways can be important at different temperatures, the tissues where they are expressed differ: heart mRNA expression explains variation in more traits at 12 °C, and brain mRNA expression explains variation in more traits at 28 °C.

## Methods

### Animal care and use

Adult fish were collected in live traps in September 2018 at three sites in central New Jersey, USA near the Oyster Creek Nuclear generating station (OCNGS). Sites included one north reference (N.Ref; 39°52′28.000 N, 74°08′19.000 W), one south reference (S.Ref; 39°47′04.000 N, 74°11′07.000 W), and a central site located within the thermal effluent of the OCNGS (TE; 39°48′33.000 N, 74°10′51.000 W). All fish were transferred live to the University of Miami, FL where they were kept in accordance with the University of Miami Institutional Animal Care and Use Committee (IACUC) guidelines.

Individuals from all three populations were common gardened to 20 °C for three months (12:12 light dark cycle) and kept at 20 °C in a common recirculating seawater system (15ppt) at 12 h light:12 h dark, then subjected to *pseudo-*winter for 6 weeks at 8 °C (8:16 light dark cycle). Following the *pseudo-winter*, half of the fish from each population were acclimated to 12 °C and the other half to 28 °C (16:8 light dark cycle) for four weeks prior to determination of WAM and CT_max_. Following this acclimation, fish originally acclimated to 12 °C were acclimated to 28 °C and vice versa for at least four weeks, and WAM and CT_max_ were measured at the new acclimation temperature. After a minimum one-week recovery period post- CT_max_, fish were sacrificed, substrate specific CaM was measured at the second acclimation temperature, and mRNA was isolated. Thus, WAM and CT_max_ were measured in all individuals at 12 °C and 28 °C, but CaM and mRNA were sampled from half the individuals acclimated at 12 °C and the other half at 28 °C.

### Quantifying metabolic and thermal tolerance traits

Individuals acclimated to 12 °C and 28 °C for at least 4 weeks were measured for whole animal metabolic rate (overnight intermittent flow respirometry) with a minimum of 20 replicate metabolic rate measures per individual used to determine the standard metabolic rate (SMR) in mgO_2_ hr^−1^ [34]. Critical thermal maximum (CT_max_) was measured in a 10-gallon tank that was slowly heated at a rate of 0.3 °C min^−1^ as in [100] and was defined as the point when fish lost equilibrium in the water column for 5 consecutive seconds. Finally, substrate specific cardiac metabolic rate (CaM, substrates: 5 mM glucose, fatty acids – 1 mM Palmitic acid conjugated to fatty-acid-free bovine serum albumin, lactate + ketones + ethanol – 5 mM lactate, 5 mM hydroxybutyrate, 5 mM ethyl acetoacetate, 0.1% ethanol, endogenous – substrate free Ringers media) was measured using micro-respirometry. Heart ventricles were dissected out and splaying in Ringers media before being transferred to a custom 1 mL chamber system [101]. Each heart was measured for glucose (GLU), then fatty acids (FA), followed by lactate + ketones + ethanol (LKA), and endogenous (END) cardiac metabolic rate. All substrates except GLU used non-reversible glycolytic enzyme inhibitors, so the order of substrates did not differ among hearts. Each heart was measured for 6 min per substrate with the last 3 min used to calculated oxygen consumption in pmolO_2_ sec^−1^. All respirometry measurements used PreSens fiber optic oxygen sensors (POF) with flow through cells (FTC-Pst7-10,WAM) or sensor spots (SP-PSt7-10, CaM) (PreSens Precision Sensing, Regensburg, Germany). Both WAM and CaM were measured at both acclimation temperatures (12 °C and 28 °C) for the same individuals, and CaM was measured at a single acclimation temperature. For additional methods and analysis of physiological data see [18].

### mRNA library preparation and sequencing

Tissues for mRNA expression were stored in chaotropic buffer at the time of CaM measurement and captured gene expression variation due to long term temperature acclimation rather than heat shock or acute temperature response (*e.g.,* resulting from higher temperatures during CT_max_ measurements) because CT_max_ measurements were performed at least a week prior to tissue isolation. We extracted total RNA from homogenized heart and brain tissues using a phenol–chloroform isoamyl alcohol isolation and treated RNA samples with DNase to remove genomic DNA. For each sample we started with 50 ng of RNA and captured the 3’ mRNA ends using an NVdT primer with a poly-A tail for first strand cDNA synthesis (Table S5). This primer contained a unique barcode for each sample (1–96), which allowed all samples in a single plate to be pooled for the remaining library preparation steps. Nick translation was used to make double stranded cDNA that was digested with an in-house purified Tn5 transposase (as in [102]) loaded with partial adapter sequences to generate fragments of double stranded cDNA ranging from ~ 300-800 bp (Table S6). Libraries were amplified for 17 PCR cycles using primers complimentary to the inserted partial adapter sequence and a plate level barcode to fully multiplex samples.

### mRNA data processing and analysis

A total of 219 libraries (110 individuals, 2 tissues per individual, 1 individual only heart was collected) were pooled and sequenced on 2 lanes of Illumina HiSeq4000 (dual end 150 bp reads) at the Genewiz LCC facility, South Plainfield NJ, USA. Raw reads were trimmed with BBDuk (from BBMap v38.87) to remove adapter sequences, aligned with STAR (v2.7.5) to the *Fundulus heteroclitus* genome, and counted with Featurecounts (v1.4.6-p5, parameters: -T 4 -s 2 -t gene -g gene_id).

The raw counts table was imported into R Studio (v1.4.1106) and all counts were normalized for library size using the median ratio method [103] with the “estimateSizeFactors” function in DESeq2 v1.6.3 [42]. Samples with a minimum of 1.5 million reads for hearts or 1 million reads for brains were retained and filtered to keep only mRNAs with at least 30 counts in 10% of individuals. After filtering, 53 heart samples and 58 brain samples remained. Principal component analysis (PCA) using “plotPCA” function from the DESeq2 package was used to examine variation among all samples. Among all samples, the 500 most variable mRNAs were used for PCA. In this analysis, 12 hearts and 13 brains were removed as outliers because they differed in expression from other same-tissue samples (*i.e.,* some hearts had “brain-like” expression patterns and vice versa*,* Fig. S2). This reduced variation within a tissue and reduced sample size to 41 hearts and 45 brains (86 total individuals); however, the individuals removed were not from a single acclimation temperature or population so likely had little overall impact on further analyses. In a separate tissue specific principal component analysis, clustering of samples by biological effects including sex, habitat temperature, population, acclimation temperature, and date of tissue collection (possible batch effect) was examined. Batch effects did not split individuals along any of the principal components examined (PC1-PC4) for heart or brain. In this separate analysis, heart PC1 accounted for 18%, heart PC2 for 7%, brain PC1 for 11%, and brain PC2 for 7% of the variation among individuals. No biologically relevant clustering was detected among the first four principal components for either tissue (chi-squared test *p* > 0.05, Fig. S3A, B). To determine the degree of variation in mRNA expression among groups within a tissue, the coefficient of variation (CV, standard deviation/mean) for each expressed mRNA was calculated and the average CV compared among groups.

### Differential expression analysis

DESeq2 v1.6.3 [42] package in R was used for differential expression analysis separately for heart and brain. To identify differentially expressed mRNAs between acclimation temperatures within populations, the DESeq model used was: ~ Population + Acclimation_Temperature + Population*Acclimation_Temperature. Additionally, due to significant interaction between population and acclimation temperature, a separate analysis was used to find differentially expressed mRNAs among populations within an acclimation temperature; individuals measured for CaM only at 12 °C or 28 °C were used with DESeq model: ~ Population. Multiple test correction across all comparisons made within a model used the Benjamin Hochberg false discovery rate with a significance threshold of 0.05.

### Weighted gene co-expression network analysis

To identify sets of co-expressed mRNAs, weighted gene co-expression network analysis (WGCNA v1.70–3, [46]) was completed for heart and brain separately. Network calculation, used to group mRNAs into co-expressed modules for heart and brain, used soft thresholding to generate a scale free network with high similarity (soft thresholding power set to 5 in heart, 4 in brain) before calculating the topological overlap measure (TOM) and using dynamicTree with minimum module size of 30 and threshold for module merging of 0.75. The first principal component of each independent module (below the threshold for module merging), known as the module eigengene (ME), was then correlated with temperature specific quantitative traits using signed Pearson’s correlation. Multiple test correction across all correlations made for a single trait used the Benjamin Hochberg false discovery rate with a significance threshold of 0.05. To remove significant correlations potentially driven by outliers, a jack-knife approach was used to subsample 90% of individuals and repeat the signed Pearson correlation analysis 100 times. Correlations that were significant in > 70 out of 100 subsamples in the same direction were robust to outliers and reported as significant. A multivariate correlation coefficient was calculated for traits significantly correlated with more than one module by correlating the fitted values from a linear model with formula: trait ~ ME1 + ME2..ME# with trait data. This multivariate correlation coefficient represents the ability of the MEs together to accurately predict the trait. For significant modules, the mRNAs with the highest module membership (MM, correlation between mRNA expression and module eigengene) and the highest gene significance for a given trait within a module (GS, correlation between mRNA expression and a given quantitative trait) were also identified.

### Gene ontology and Kyoto encyclopedia of genes and genomes enrichment

To identify biologically important networks within WGCNA modules that were significantly correlated with at least 1 trait, we used KEGG pathway and GO enrichment analyses. First, the genome was mapped to KEGG and GO terms using eggNOG mapper [104] with default parameters. The KEGG and GO terms were then matched to the set of expressed mRNAs for heart and brain. The list of mRNAs in each module was compared to the set of expressed mRNAs (set as the reference or gene universe) in each tissue for enrichment analysis in R using the clusterProfiler v3.16.1 package “enricher” function for KEGG terms [105]. To map enriched KEGG terms to KEGG pathways, the KEGG Mapper online tool was used with annotations from the closest relative, zebrafish (*Danio rerio*) [106, 107]. Cytoscape v3.8.2 with BiNGO v3.0.3 was used for GO enrichment using the set of expressed mRNAs as the reference to examined enrichment of biological process, molecular function, and cellular component GO terms [108]. Significant KEGG and GO terms are reported with FDR *p*-value threshold of 0.05.

## Supplementary Information

**Additional file 1:** **Figure S1.** Trait variance in metabolic rate among ectotherms. A) Standard metabolic rate variance among species from different climates are compared to average variance in metabolic rate within the Fundulus heteroclitus populations used in this study. Variance calculated as mass corrected maximum (in warmer environment) – minimum (in cooler environment)/minimum (in cooler environment) (range spread).All standard metabolic rates are corrected for body mass (residual of metabolic rate vs. body mass + metabolic rate of an average sized fish from this data set). B) Number of species per group. Data from (1). **Figure S2.** Principal component analysis of all samples. Principal component 1 split heart and brain tissue and explained 86% of variance. Individuals who did not clearly group with the appropriate tissue were removed as outliers. **Figure S3.** Tissue specific principal component analysis. Heart (*N*=41, A) first two principal components explain 19% and 7% of variance. Brain (*N*=45, B) first two principal components explain 11% and 7% of variance. Triangles are 28°C acclimated individuals, circles are 12°C. Individuals from the north reference (N.Ref) are blue, south reference (S.Ref) are purple, and thermal effluent population (TE) are red. **Figure S4.** Differentially expressed mRNAs among populations within tissue and acclimation temperature. A) Heart at 12°C, B) brain at 12°C, C) heart at 28°C, D) brain at 28°C. Populations are north reference (N.Ref), south reference (S.Ref), and thermal effluent (TE).

**Additional file 2:** **Table S1.** Significant Acclimation effect on mRNA Expression within each Population. Population differential expression.with FDR *p*<0.05.  Differentially expressed genes among population pairs within 12°C or 28°C with FDR *p*<0.05. **Table S2.** Population differential expression for each acclimation temperature with FDR <0.05 . Differentially expressed genes between 12°C and 28°C within populations with FDR *p*<0.05.Temperature differential expression. Differentially expressed genes between 12°C and 28°C within populations with FDR *p*<0.05. **Table S3.** Enriched Kyoto Encyclopedia of Genes and Genomes (KEGG) Pathways within Significant Modules. Module identifier (Module), correlated trait(s), enriched KEGG terms, and KEGG pathways are listed in columns. Enriched KEGG terms with FDR *p*-value < 0.05 were mapped to KEGG pathways using KEGG Mapper. Terms of interest based on relationship to correlated trait(s) are underlined. **Table S4.** Enriched Gene Ontology (GO) Terms within Significant Modules. Modules are named based on the tissue they are in and trait they are significantly correlated with. Enriched GO terms with FDR *p*-value < 0.05 are listed. Terms of interest based on relationship to correlated trait(s) are underlined. **Table S5.** Primers and barcodes for 3’ mRNA library preparation. First strand synthesis with i7 primers added i7 indicies. Second strand synthesis added i5 index to 96 pooled samples. **Table S6.** Tn5 loading and PCR amplification primer sequences. The lower-case nucleotides complement the Tn5MErev primer. The upper-case underlined nucleotides match with the i5 (R1) and P7 match with the i7 (part of the NVdT primer). 

## Data Availability

All sequence data is available in NCBI SRA: https://dataview.ncbi.nlm.nih.gov/object/PRJNA796010?reviewer=f1u5dbo46558lklv8m2cknap87 (public DOI available upon publication). All physiological data is available in Dryad: https://doi.org/10.5061/dryad.0gb5mkm0w. Code for processing of raw sequence files and all data analysis and visualization conducted in R is available in Github: https://github.com/mxd1288/OCNJ_F18_RNA.git.
