# Supplementary material for: Transcriptomic analysis provides insights into molecular mechanisms of thermal physiology
Source: BMC Genomics. 2022 Jun 4;23:421. doi: 10.1186/s12864-022-08653-y (PMC9167525; doi:10.1186/s12864-022-08653-y)
Supplement: Supplementary file 1 — Additional file 1: Figure S1. Trait variance in metabolic rate among ectotherms. A) Standard metabolic rate variance among species from different climates are compared to average variance in metabolic rate within the Fundulus heteroclitus populations used in this study. Variance calculated as mass corrected maximum (in warmer environment) – minimum (in cooler environment)/minimum (in cooler environment) (range spread).All standard metabolic rates are corrected for body mass (residual of metabolic rate vs. body mass + metabolic rate of an average sized fish from this data set). B) Number of species per group. Data from (1). Figure S2. Principal component analysis of all samples. Principal component 1 split heart and brain tissue and explained 86% of variance. Individuals who did not clearly group with the appropriate tissue were removed as outliers. Figure S3. Tissue specific principal component analysis. Heart (N=41, A) first two principal components explain 19% and 7% of variance. Brain (N=45, B) first two principal components explain 11% and 7% of variance. Triangles are 28°C acclimated individuals, circles are 12°C. Individuals from the north reference (N.Ref) are blue, south reference (S.Ref) are purple, and thermal effluent population (TE) are red. Figure S4. Differentially expressed mRNAs among populations within tissue and acclimation temperature. A) Heart at 12°C, B) brain at 12°C, C) heart at 28°C, D) brain at 28°C. Populations are north reference (N.Ref), south reference (S.Ref), and thermal effluent (TE). [file 12864_2022_8653_MOESM1_ESM.docx]

**Figure S1: Trait variance in metabolic rate among ectotherms. A)** Standard metabolic rate variance among species from different climates are compared to average variance in metabolic rate within the *Fundulus heteroclitus* populations used in this study. Variance calculated as mass corrected maximum (in warmer environment) – minimum (in cooler environment)/minimum (in cooler environment) (range spread).All standard metabolic rates are corrected for body mass (residual of metabolic rate vs. body mass + metabolic rate of an average sized fish from this data set). **B)** Number of species per group. Data from (1).

**Figure S2: Principal component analysis of all samples.** Principal component 1 split heart and brain tissue and explained 86% of variance. Individuals who did not clearly group with the appropriate tissue were removed as outliers.

**Figure S3: Tissue specific principal component analysis.** Heart (N=41, A) first two principal components explain 19% and 7% of variance. Brain (N=45, B) first two principal components explain 11% and 7% of variance. Triangles are 28°C acclimated individuals, circles are 12°C. Individuals from the north reference (N.Ref) are blue, south reference (S.Ref) are purple, and thermal effluent population (TE) are red.

**
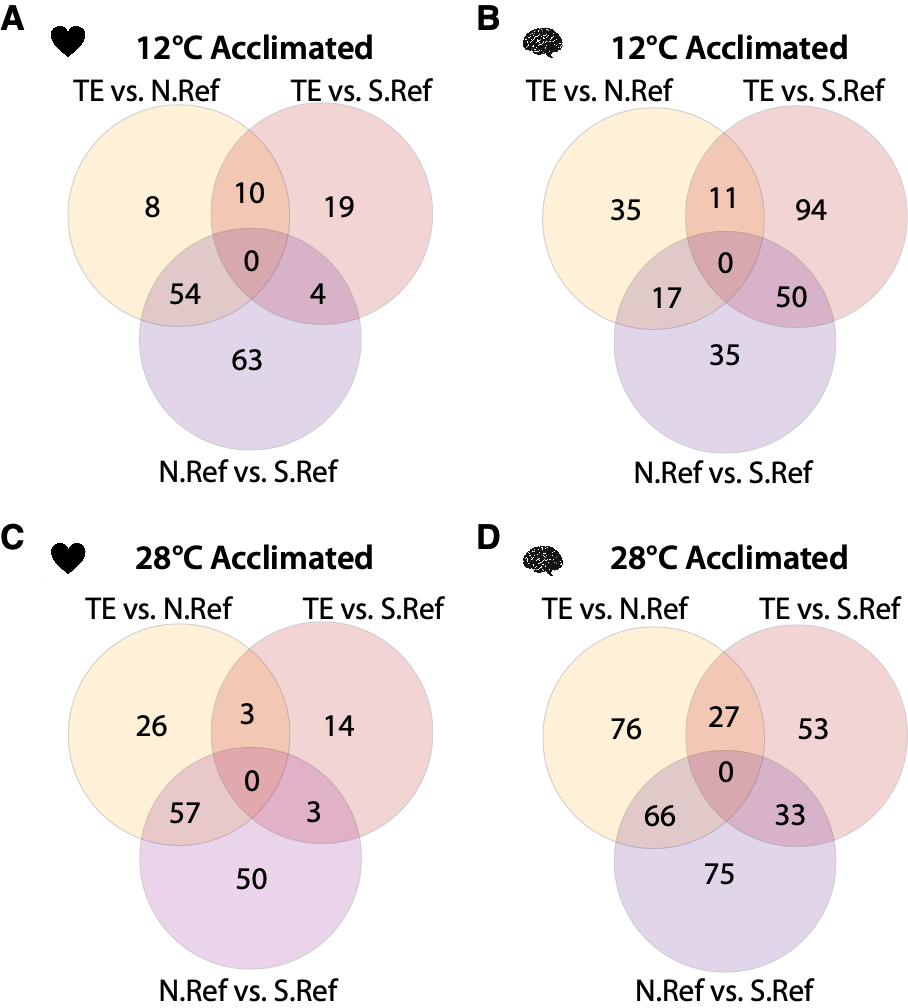
**

**Figure S4: Differentially expressed mRNAs among populations within tissue and acclimation temperature. A)** Heart at 12°C, **B)** brain at 12°C, **C)** heart at 28°C, D) brain at 28°C. Populations are north reference (N.Ref), south reference (S.Ref), and thermal effluent (TE).
